# Design of the first national lung cancer screening program in the European Union: the Croatian Model

**DOI:** 10.1007/s00330-025-12185-w

**Published:** 2025-12-06

**Authors:** Miroslav Samaržija, Kristina Krpina, Ante Marušić, Marko Jakopović, Antun Aboud, Melita Kukuljan, Vjekoslava Amerl Šakić, Ines Balint, Hans-Ulrich Kauczor, Rowena Yip, David Yankelevitz, Claudia Henschke

**Affiliations:** 1 University Hospital Centre Zagreb, Department for Respiratory Disease, Jordanovac 104, Zagreb, Croatia; 2 University of Split, Department for Forensic Sciences, Split, Croatia; 3 University Hospital Centre Rijeka, Department for Radiology, Rijeka, Croatia; 4 General Practice Zagreb, Zagreb, Croatia; 5 University Hospital Heidelberg, German Center of Lung Research, Diagnostic and Interventional Radiology, Heidelberg, Germany; 6 Department of Diagnostic, Molecular, and Interventional Radiology, Icahn School of Medicine at Mount Sinai, New York, NY USA

**Keywords:** Early detection of Cancer, Mass screening, Lung neoplasms, National Health Programs, Tomography (X-ray computed)

## Abstract

**Objectives:**

To address Croatia’s high lung cancer mortality and late-stage diagnoses, the Ministry of Health initiated a multidisciplinary effort to design a national lung cancer screening program.

**Materials and methods:**

Lung cancer remains one of the leading causes of cancer-related mortality both globally and in Croatia. In 2021 alone, Croatia recorded over 3300 new cases of lung cancer and more than 2800 associated deaths, indicating a high mortality burden. In response to this public health concern, the Ministry of Health has established a multidisciplinary Lung Cancer Screening Working Group, tasked with developing a national screening approach. The Program incorporates several innovative elements, including the application of modified International Early Lung Cancer Action Program (I-ELCAP) criteria for nodule management, volumetric analysis assessed by artificial intelligence, complete digitalization, smoking cessation, and nationwide deployment to ensure equitable access.

**Results:**

From October 2020 to August 2025, over 50,000 participants were screened, resulting in more than 70,000 LDCT scans performed. The cohort includes 54% male and 46% female participants, with an average age of 62 years. Among these participants, 4.5% had positive results, which required further follow-up.

**Conclusion:**

The Croatian National Lung Cancer Screening Program offers unique features as it has been comprehensively incorporated into the existing healthcare infrastructure and is fully reimbursed. A key aspect of the program is the important role assigned to general practitioners (GPs), who are responsible for identifying and referring individuals at high risk for lung cancer.

**Key Points:**

***Question*** *No European Union country has implemented a national lung cancer screening program despite evidence from previous trials showing significant mortality reduction.*

***Findings*** *Croatia successfully launched a fully integrated national lung cancer screening program using LDCT, AI-assisted volumetric analysis, modified I-ELCAP criteria, and GP-centered recruitment.*

***Clinical relevance*** *The Croatian model demonstrates the feasibility of national lung cancer screening within a European public healthcare system with full reimbursement, providing a replicable framework for other EU countries implementing lung cancer screening programs.*

**Graphical Abstract:**

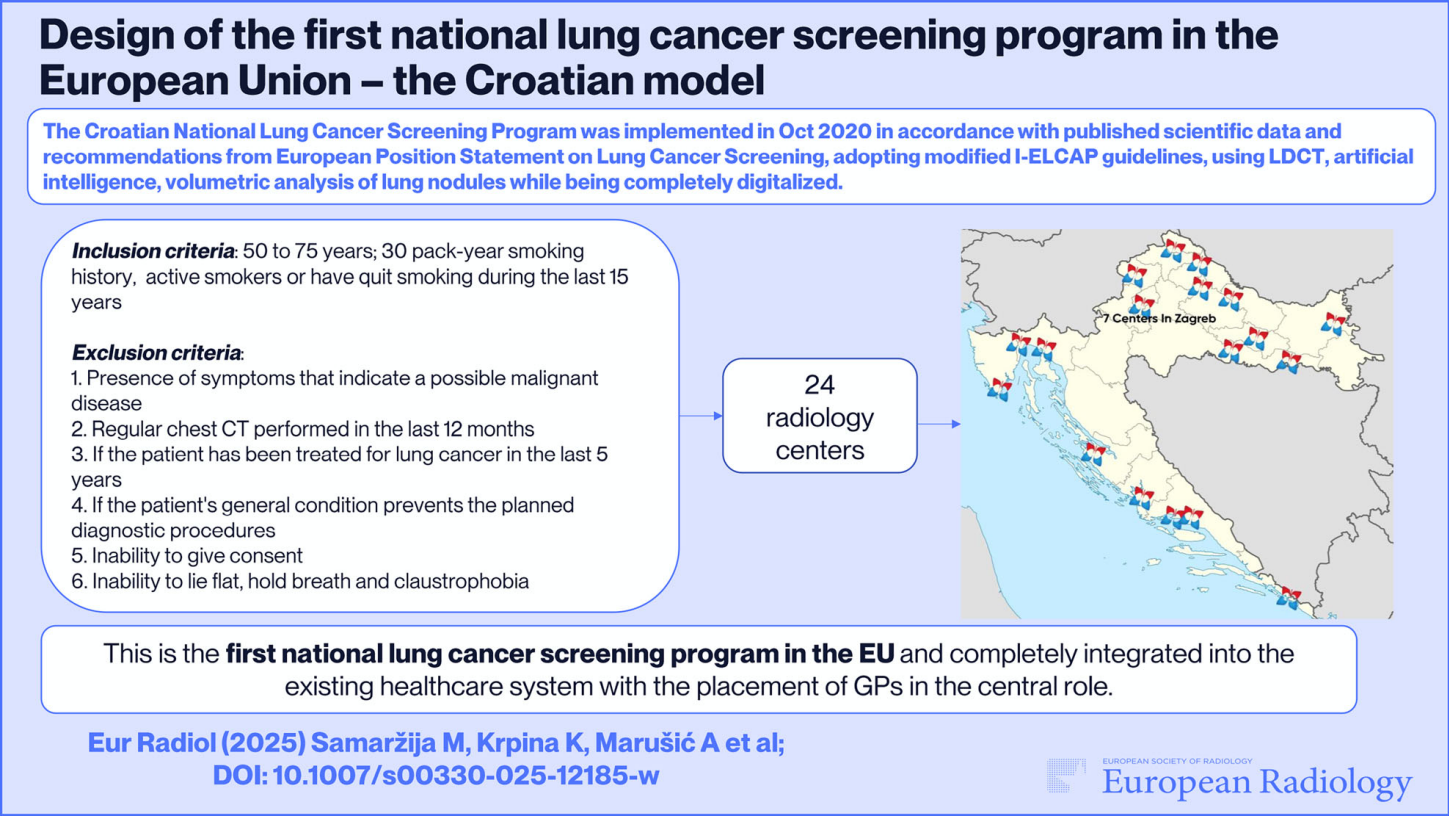

## Introduction

Lung cancer is one of the leading causes of death from malignant diseases, both worldwide and in Croatia [1–9]. With more than 3300 new diagnoses of lung cancer and more than 2800 lung cancer deaths in 2021 on a population of 3879 million, lung cancer remains one of the main public health problems in Croatia [4–9]. For more than a decade, population-based screening programs for breast, colorectal and cervical cancers have been implemented in the Western world and in Croatia, despite lung cancer being the leading cause of cancer-related deaths, more than the above-mentioned cancers combined [9]. This puts into perspective the importance of early detection and screening for lung cancer (LCS). Regardless of recent advances in lung cancer treatment, the overall five-year survival rate remains low, with only approximately 15% of patients surviving beyond five years following their initial diagnosis [1]. Data for Croatia show a 10% 5-year survival rate, while the same data for the European Union (EU) is 15%, and it has one of the highest mortality-to-incidence ratios in the EU. One of the main reasons is that approximately two-thirds of patients are diagnosed at advanced stages when surgical treatment is not an option, leaving only systemic treatments available.

Although the prevalence of smoking among adults is decreasing in the Western world, it remains high in Croatia. Tobacco consumption remains a leading risk factor for developing lung cancer. This means that smokers and ex-smokers are at higher risk for developing lung cancer, which was the reason that previous screening trials included only smokers and ex-smokers [7].

### Evidence from NLST and NELSON

The first, and to date, the largest lung cancer screening trial was conducted in the United States. The National Lung Screening Trial (NLST) enrolled 53,454 participants aged 55 to 74 years at high risk for lung cancer [10]. The strategy of three annual low-dose computed tomography (LDCT) screenings led to a 20% reduction in lung cancer mortality compared to chest X-ray screening after a median follow-up of more than six years [11]. The high-risk population was defined as individuals aged 55 to 74 years with a 30-pack-year history of smoking who were either current smokers or had quit smoking within the previous 15 years. However, at the time of designing the Croatian lung cancer screening program, despite strong and convincing data, only about 4% of eligible high-risk persons have been screened in the United States after the introduction of the screening program provided by healthcare insurance programs [12]. This has been attributed to patient anxiety about potential work-up, which included different types of biopsies (CT-guided, bronchoscopy, surgical), PET-CT scans, and potential surgical procedures.

The results of the European Nederlands-Luevens Longkanker Screenings Onderzoek (NELSON) trial were first presented in 2018 [13] and published in 2020 [14]. NELSON is a Dutch-Belgian, population-based, randomized lung cancer screening trial initiated in 2000. The study was designed for a high-risk male population with volume-based LDCT to assess a decrease in mortality over a 10-year follow-up period with LDCT scheduled at baseline, 1 year, 3 years, and 5.5 years [14]. However, amendments to the study were made to include high-risk females as well, due to an increase in the number of female smokers, as well as an increase in incidence and mortality from lung cancer in females.

The at-risk population was defined as persons aged 50 to 74, who were current smokers or former smokers (who quit within the past 10 years) with a history of smoking more than 15 cigarettes per day for over 25 years, or more than 10 cigarettes per day for over 30 years. In this trial, participants were randomized into two groups: screening with low-dose CT compared to no screening. After ten years of follow-up, the cumulative risk reduction of lung cancer mortality was 24% in the screening group. Among the females, the NELSON trial observed a risk reduction of lung cancer mortality of 33%. The study also reported low rates of follow-up procedures for LDCT scans [14].

Robust evidence from relevant published data, especially the NLST and NELSON trials, has shown the effectiveness of low-dose CT screening as a preferred method for identifying persons at higher risk of developing lung cancer. Based on that data, the European Position Statement on Lung Cancer Screening was presented in 2017, and concluded that LDCT is the best method for lung cancer screening. The statement concluded that planning for the implementation of LDCT screening should begin across Europe as soon as possible, and that EU countries should set a timeline for implementing lung cancer screening [15].

Despite convincing data and recommendations from the European Position Statement on Lung Cancer Screening, none of the countries in the EU had implemented a national lung cancer screening program (NLCSP) [11, 13, 16, 17]. Recognizing this gap and considering the high prevalence of smoking among adults, poor incidence to mortality ratio, and high incidence of diagnosis of lung cancer in advanced stages in Croatia, the lung cancer screening working group, established by the Ministry of Health, launched the NLCSP in Croatia in 2020, the first government-funded NLCSP program in Europe. This paper describes the design and implementation framework of the Croatian NLCSP.

## Materials and methods

Croatian National Lung Cancer Screening Program (NLCSP) offers several unique features, such as modified I-ELCAP criteria, GPs in the central role of the integrated approach, complete digitalization, smoking cessation programs, and nationwide application. While many studies have explored ways to detect lung cancer at an early stage, two large, randomized trials, the NLST and NELSON, showed a clear reduction in mortality and became the foundation for the Croatian National Lung Cancer Screening Program [10, 11, 13, 14, 18, 19].

### Eligibility criteria

In Croatia, the NLCSP includes persons from 50 to 75 years with a 30 pack-year smoking history who are active smokers or have quit smoking during the last 15 years. Exclusion criteria are listed below:

1. The presence of symptoms that indicate a possible malignant disease
2. Regular chest CT performed in the last 12 months
3. If the patient has been treated for lung cancer in the last 5 years
4. If the patient’s general condition prevents the planned diagnostic procedures
5. Inability to give consent
6. Inability to lie flat, hold breath, and claustrophobia

### Screening protocol

Published data show that different screening trials have used different protocols for work-up, recommendations, and resulting diagnoses of lung cancer. At the time of designing the Croatian National LC Screening Program, there were three published protocols: I-ELCAP, ACR Lung-RADS (American College of Radiology—Lung CT Screening Reporting and Data System) and European Consortium [15, 20–24]. All three protocols measure solid, non-calcified nodules (NCNs)in full, but differ in part-solid NCNs measurement. For part-solid nodules, I-ELCAP uses the diameter of the solid component, while ACR Lung-RADS uses both the entire diameter of the part-solid nodules as well as the diameter of their solid component. The European Consortium protocol determines the volume of solid NCNs using its proprietary software. Given that volumetric measurements for entire part-solid and non-solid NCNs are problematic, the European Consortium protocol specifies the equivalent diameter values for each. Each protocol provides threshold values for its work-up. Threshold mean diameter values are as follows: for I-ELCAP, 6.0 and 15.0 mm; for ACR Lung-RADS, 6 mm, 8 mm, and 15 mm; and for the European Consortium, 50 mm^3^ (equivalent to 5 mm) and 300 mm^3^ (10 mm) for solid NCNs, and 5 mm for part-solid and non-solid NCNs.

The three most widely adopted protocols for lung cancer screening work-up, the I-ELCAP, ACR Lung-RADS, and European Consortium guidelines, were directly compared by Henschke et al [25]. Each of these protocols follows a stepwise approach. The initial LDCT scan is used to detect large or advanced cancers requiring immediate intervention, while subsequent LDCTs focus on monitoring smaller NCNs for potential growth indicative of malignancy. In their comparative analysis, Henschke et al reported the proportion of positive screening results as follows: 1.4% for I-ELCAP, 5.9% for ACR Lung-RADS (reduced to 2.1% when Scenario 2 criteria were applied), and 3.1% for the European Consortium protocol [25]. These findings highlight significant differences in sensitivity and specificity among the protocols, which may influence clinical decision-making and patient outcomes.

### Modified I-ELCAP protocol for the Croatian Program

Based on these findings, the I-ELCAP protocol [22] demonstrated the highest efficiency in terms of immediate work-up, overall diagnostic management, and biopsy recommendations. Consequently, a modified version of the I-ELCAP criteria was adopted for implementation within the Croatian National Lung Cancer Screening Program (Table 1). The modified protocol incorporates additional elements, including volume-based assessment of lung nodules and refined categorization of imaging findings, to enhance diagnostic accuracy and optimize clinical decision-making. All modifications were carried out in accordance with the approval of the I-ELCAP clinical team. At the time of designing the Croatian Program, the original protocol was based on the average nodule diameter. Volume measurements were introduced to align nodule size with diameters. Accordingly, a nodule follow-up recommendation based on volume doubling time was introduced and aligned with the I-ELCAP protocol, which was based on diameter change. Classification of the results into categories provides a better understanding of the small nodule findings. Biennial screening is conducted for participants with negative baseline and regular follow-up LDCT with no new or growing nodules, reducing unnecessary exposure and resource use, following I-ELCAP guidelines.

**Table 1:** Modified I-ELCAP recommendations for handling the pulmonary nodules in the national screening program for lung cancer in Croatia

Baseline LDCT	Regular annual/biennial LDCT	Follow-up LDCT
**a)** **NEGATIVE:** **1) If there are NO non-calcified nodules (NCNs)**. *Recommendation: Return for the first annual screening in 12 months*	**a)** **NEGATIVE:** **1) There are NO NEW or GROWING nodules**. *Recommendation: Next annual repeat screening in 24 months*	**a)** **NEGATIVE:** **1) Nodule resolved** *Recommendation: Next annual repeat screening in 24 months*
**b)** **SEMI-POSITIVE (OR INDETERMINATE):** **1) Only non-solid nodules are present; they can be of any size**. *Recommendation: Return for the first annual screening in 12 months* **2) Largest solid NCN** < **6.5 mm (volume** < **150.0 mm**^**3**^**) or largest solid component of a part-solid NCN** < **6.5 mm (volume** < **150.0 mm**^**3**^**)**. *Recommendation: Return for the first annual screening in 12 months* **3) Largest NCN is solid** ≥ **6.5 mm in average diameter (volume** ≥ **150.0 mm**^**3**^**) OR** **largest NCN is part-solid and the solid component** ≥ **6.5 mm in average diameter (volume** ≥ **150.0 mm**^**3**^**), but** < **15.5 mm (2000 mm**^**3**^**)** *Recommendation: Return for LDCT in three (3) months* **4) Endobronchial solid of any size** *Recommendation: Return for LDCT in one (1) month*	**b**) **SEMI-POSITIVE (OR INDETERMINATE):** **1**) **Only NEW or SLOWLY GROWING non-solid NCNs of any size**. *Recommendation: Return for next annual repeat LDCT screening in 12 months*. **2**) **Largest solid NEW or GROWING NCN OR solid component of NEW or GROWING part-solid NCN is** < **3.0 mm in average diameter, (volume of 20.0 mm**^**3**^**)**. *Recommendation: Return for next annual repeat LDCT screening in 12 months* **3**) **Largest NEW OR GROWING solid NCN OR solid component of NEW or GROWING part-solid NCN** **is between 3.0–6.5 mm in average diameter (Volume between 20.0 mm**^**3**^ **and 150.0 mm**^**3**^**)**. *Recommendation: Return for LDCT in 6 months* **4**) **Largest NEW or GROWING solid NCN** ≥ **6.5 mm in average diameter (Volume** ≥ **150 mm**^**3**^) **OR** **largest NEW or GROWING solid component of a part-solid NCN** ≥ **6.5 mm in average diameter (Volume** ≥ **150 mm**^**3**^**)**. *Recommendation: Return for LDCT in 1 month and consider antibiotics prior to 1-month LDCT* **5**) **New endobronchial nodule of any size** *Recommendation: Return for LDCT in one (1) month*	**b)** **SEMI-POSITIVE (OR INDETERMINATE):** **1) VDT** ≥ **600 days** *Recommendation: Return for next annual repeat LDCT screening at 12 months from baseline (annual screening)* **2) VDT 400–600 days** *Recommendation: Return for 1st repeat LDCT screening in 6 months, if growth at the same rate, repeat CT in 12 months*
**c)** **POSITIVE:** **1) Largest solid NCN** ≥ **15.5 mm (volume** ≥ **2000.0 mm**^**3**^**)** *Recommendation: Referral to screening pulmonologist/nodule clinic*	**c)** **POSITIVE:** **1) Non-solid nodule doubled in volume in 1 year (suggest only if a solid component emerges)** **2) Persistent endobronchial nodule after 1 month** *Recommendation: Referral to screening pulmonologist/nodule clinic*	**c)** **POSITIVE:** **1) VDT** < **400 days** *Recommendation: Referral to screening pulmonologist/nodule clinic* **2) Persistent endobronchial nodule** *Recommendation: Referral to screening pulmonologist/nodule clinic* **3) VDT 400–600 days** *Recommendation: When larger than 15 mm (2000 mm*^*3*^*), refer to the screening pulmonologist/nodule clinic*
**a.1**. 12 months	**a.1**. 24 months	**a.1**. 24 months
**b.1 & 2**. 12 months	**b.1 & 2**. 12 months **b.3**. 6 months **b.4**. 1 month + antibiotic **b.5**. 1 month	**b.1**. in 6 or 9 months–at 12 months (annual screening) **b.2**. 1st 6 months, 2nd 12 months
**b.3**. 3 months		
**b.4**. 1 month		
**c.1**. Screening pulmonologist/nodule clinic	**c.1. & 2**. Screening pulmonologist/nodule clinic	**c.1. & 2. & 3**. Screening pulmonologist/nodule clinic

### Imaging requirements

The LDCT scanning is currently available at 24 sites (17 cities across the country) with plans for expansion. The minimum technical requirements for LDCT units are given below in Table 2:

**Table 2:** Minimum technical requirements for LDCT scanners

**1**.	Peak kilovoltage	120–140 kVp
**2**.	Tube current	20–60 mAs
**3**.	Collimation	=< 1 mm
**4**.	Gantry rotation time	=< 0.5 s
**5**.	CTDIvol	=< 3.0 mGy
**6**.	Average effective dose	=< 1.5 mSv
**7**.	Multidetector CT	>= 128 detector slices
**8**.	Image slice thickness	=< 1 mm

As more suitable CT units become available, the network of radiology sites will be expanded accordingly. The goal of the low-dose scanning is to achieve less than 1.5 mSv effective radiation dose per scan. The radiologists have received additional training and have been licensed for the special skills needed to report screening scans correctly, using the Modified I-ELCAP Croatia protocol (Table 1).

### Radiologist training and licensing

Licensing of radiologists is achieved after completing a course that covers lectures, workshops on lung nodules and software possibilities, and patient cases. Only experienced radiologists (more than 300 thorax CT scans per year) from high-volume thoracic centers can be licensed. Introductory training courses have been organized on a regular basis for all newly participating radiologists. Each course spans a full day and is structured into two parts: a session devoted to lectures, followed by a hands‑on session. The practical component includes the review of approximately 30 representative cases, which are jointly discussed at the conclusion of the session. A volume measurement tool is available during the training to support standardized assessment and familiarization with the workflow.

### Digitalization

There are several unique features of the program. For the purpose of the NLCSP, a unique IT application has been developed and incorporated into the existing national IT healthcare platform, providing a secure and completely paperless system for radiology and pulmonology referrals, structured reporting, and report sharing. The advantage of such an IT solution is an integrated care approach and central data storage, which serves a national lung cancer screening register. The NLCSP software uses a unique identifier for each screening participant, in the form of a health insurance number, which also functions as a personal identifier for all other healthcare procedures. All data is shared with and stored in the patient’s digital personal healthcare record. This digital infrastructure enables efficient communication between primary care physicians, radiology centers, and specialized pulmonology clinics, significantly reducing delays in diagnosis and treatment initiation.

### Multidisciplinary collaboration in workflow and integration

#### Role of general practitioners

A major difference when compared to other programs is the placement of general practitioners (GPs) in a central role to enrol the high-risk population without sending out invitation letters. In Croatia, there is a network of approximately 2300 GP clinics throughout the country taking care of about 1800 patients each. Each person in Croatia has a dedicated GP, and approximately 90% of the population visits their GP during one calendar year [26]. As mentioned above, the conclusion is that every GP can expect at least one visit during the year from individuals eligible for NLCSP and can conduct motivational interviews on that occasion. Therefore, the NLCSP starts at the GP office with GPs checking for eligible candidates (inclusion and exclusion criteria) during dedicated or non-dedicated visits. The GPs can also search for screening candidates actively in their digital archive, where they have stored data on the smoking history of their patients. After identifying the individual, the GP should conduct a motivational interview with potential screening candidates. Should the eligible person decide to become an NLCSP participant, their GP will be able to enrol them into the program, which includes smoking cessation counseling, a precise description of possible harms and benefits of the program, as well as choose from available appointments for LDCT scanning at any of the 24 radiology sites across the country based on location and appointment availability. To combat the low rate of eligible persons who respond to screening programs, as has occurred in the United States study (response below 4%), the role of the GP was positioned at the center of the program, having in mind the benefit of a good doctor-patient relationship. This should significantly increase the number of persons who will enter the program after being identified, but also improve the response rate to LDCT screening.

Upon entering the program, the participant is presented with additional information about the program, the harms and benefits of LDCT, as well as smoking cessation support and assistance possibilities. At the radiology department, the patient must sign the informed consent form before undergoing an LDCT scan. Part of the informed consent form is related to further data use for academic and research purposes.

#### Role of radiology and pulmonology

The NLCSP is offered after hours, which reduces the health hazard for the participants and does not interfere with regular waiting lists. For radiologists and GPs, the NLCSP is treated as extra work with an additional financial incentive.

A licensed radiologist reads the scan and performs volumetric analysis and lung nodule detection utilizing clinical applications incorporating artificial intelligence (AI) for multi-modality reading. The radiologist’s report is structured and includes detected nodules as well as additional findings, such as emphysema, coronary artery calcification and the diameter of the aorta and pulmonary artery. For coronary artery calcification and emphysema, which are classified in four stages as none, mild, moderate and severe, a visual scale is used. The Modified I-ELCAP Croatia protocol is used to categorize the lung nodules and give recommendations for further LDCT follow-up or referral to the screening lung nodule clinic.

The structured radiology report is available to the GP along with precise instructions on the further steps to take. In the case of annual or biennial regular follow-up LDCT or the need for early recall, the GP will submit another electronic referral to one of the radiology sites in cooperation with the screening participant. In the case of a positive LDCT result, the GP submits an electronic referral within the application to one of 6 screening pulmonology sites – nodule clinics. In the case of significant additional findings, the GP makes a regular referral for further workup at the appropriate institutions.

The aim of NLCSP is to offer quick and comprehensive management of suspected lung cancers at lung nodule clinics that are established in 5 cities (6 sites) across the country. Management includes a multidisciplinary approach, complete diagnostic work-up (bronchoscopy, CT-guided biopsies or fine needle aspirations, CTs, PET scans or MRIs), as well as thoracic surgery and on-site departments for personalized oncology treatments. The purpose of lung nodule clinics is to enable an early diagnostic and treatment approach to patients with suspected lung cancer in a timely manner and to increase the cure rate in this group.

In the lung nodule clinic, the screening participant is interviewed and examined by a pulmonologist. If the first pulmonology report confirms the suspicion of lung cancer and the need for further work-up, the screening participant becomes a patient. After 6 months, the pulmonologist is obliged to issue the final medical report using the NLCSP digital application, which includes the diagnosis (pathohistological diagnosis), staging (tumor, node, metastasis staging system) and applied treatment.

### Quality assurance and control

Quality control is an important part of the NLCSP, which includes the involvement of domestic and international quality control committees, regular reports on radiation dose, reports on the number of participants and detailed demographic data, as well as radiology and pulmonology reports, all generated within the NLCSP application. Apart from regular supervision from local medical physicists, the LDCT units check regularly for radiation exposure on phantoms by an independent institution, i.e., the Unit of Radiation Dosimetry and Radiobiology at the Institute for Medical Research and Occupational Health, using standardized phantoms. The structured radiology report has a mandatory field for effective radiation dose per scan and a cumulative dose for each screening participant. The basic LDCT scanning protocols are aligned with guidelines of the American Association of Physicists in Medicine [27], but are under continuous evaluation and improvement by local medical physicists with the support and guidance of the Croatian Medical Physicists Association.

## Results

From October 2020 to August 2025, over 50,000 participants were screened, resulting in more than 70,000 LDCT scans performed. The cohort includes 54% male and 46% female participants, with an average age of 62 years. Among these participants, 4.5% had positive results. Among those participants with a positive result, 2% of cancers were found (1.7% lung cancer). Detailed analysis of outcomes in this referral group will be reported separately, and further analyses are ongoing.

All participating radiology sites met or exceeded radiation safety standards. The mean effective dose was 0.89 mSv, markedly below the maximum limit of 1.5 mSv set at the beginning of the program.

## Conclusion

The Croatian NLCSP is the first NLCSP in Europe that is completely integrated into an existing healthcare system and fully covered by the National Health Insurance Fund. The design of the Croatian National Lung Cancer Screening Program reflects a comprehensive and multidisciplinary approach to implementing a national screening strategy. This structured framework lays a strong foundation for ongoing evaluation, positioning Croatia as a model for lung cancer screening programs within Europe.

## Abbreviations

ACR Lung-RADS: American College of Radiology—Lung CT Screening Reporting and Data System
CT: Computed tomography
EU: European Union
GP: General practitioner
I-ELCAP: International Early Lung Cancer Action Program
IT: Information technology
LDCT: Low-dose computed tomography
mSv: Millisievert
NCN: Non-calcified nodule
NELSON: Nederlands-Luevens Longkanker Screenings Onderzoek
NLCSP: National Lung Cancer Screening Program
NLST: National Lung Screening Trial
PET-CT: Positron emission tomography computed tomography

## References

[1] European Commission (2025) European Cancer Information System—Data Explorer. Available via https://ecis.jrc.ec.europa.eu/data-explorer#/. Accessed 24 Apr 2025

[2] World Health Organization (2025) WHO Mortality Database. Health statistics and information systems. Available via https://www.who.int/data/data-collection-tools/who-mortality-database. Accessed 24 Apr 2025

[3] Bray F, Laversanne M, Sung HYA et al (2024) Global cancer statistics 2022: GLOBOCAN estimates of incidence and mortality worldwide for 36 cancers in 185 countries. CA Cancer J Clin74:229–263 38572751 10.3322/caac.21834

[4] Ferlay J, Colombet M, Soerjomataram I et al (2018) Cancer incidence and mortality patterns in Europe: Estimates for 40 countries and 25 major cancers in 2018. Eur J Cancer 103:356–387 30100160 10.1016/j.ejca.2018.07.005

[5] Baum P, Winter H, Eichhorn ME et al (2022) Trends in age- and sex-specific lung cancer mortality in Europe and Northern America: Analysis of vital registration data from the WHO Mortality Database between 2000 and 2017. Eur J Cancer 171:269–279 35738973 10.1016/j.ejca.2022.05.011

[6] Eurostat Cancer statistics (2025) Available via https://ec.europa.eu/eurostat/statistics-explained/index.php?title=Cancer_statistics#Deaths_from_cancer. Accessed 26 Mar 2025

[7] OECD (2025) EU Country Cancer Profile: Croatia 2025. Available via https://www.oecd.org/content/dam/oecd/en/publications/reports/2025/02/eu-country-cancer-profile-croatia-2025_dc412e98/46c5e70c-en.pdf. Accessed May 2 2025

[8] Croatian Institute of Public Health (2021) Cancer Incidence in Croatia 2019—Bulletin No. 44. Available via https://www.hzjz.hr/wp-content/uploads/2021/12/Bilten44_2019.pdf. Accessed 2 May 2025

[9] Croatian Institute of Public Health (2020) Cancer Incidence in Croatia 2017—Bulletin. Available via https://www.hzjz.hr/wp-content/uploads/2017/01/Bilten-2017-final.pdf. Accessed 2 May 2025

[10] National Lung Screening Trial Research Team, Aberle DR, Berg CD et al (2011) The National Lung Screening Trial: overview and study design. Radiology 258:243–253 21045183 10.1148/radiol.10091808 PMC3009383

[11] National Lung Screening Trial Research Team, Aberle DR, Adams AM et al (2011) Reduced lung-cancer mortality with low-dose computed tomographic screening. N Engl J Med 365:395–409 21714641 10.1056/NEJMoa1102873 PMC4356534

[12] Okereke IC, Nishi S, Zhou J, Goodwin JS (2019) Trends in lung cancer screening in the United States, 2016-2017. J Thorac Dis 11:873–881 31019776 10.21037/jtd.2019.01.105 PMC6462682

[13] De Koning H, Van der Aalst C, Ten Haaf K, Oudkerk M (2018) Effects of volume CT lung cancer screening: mortality results of the NELSON randomised-controlled population based trial. J Thorac Oncol 13:S185

[14] de Koning HJ, van der Aalst CM, de Jong PA et al (2020) Reduced lung-cancer mortality with volume CT screening in a randomized trial. N Engl J Med 382:503–513 31995683 10.1056/NEJMoa1911793

[15] Oudkerk M, Devaraj A, Vliegenthart R et al (2017) European position statement on lung cancer screening. Lancet Oncol 18:e754–e766 29208441 10.1016/S1470-2045(17)30861-6

[16] Kauczor HU, Baird AM, Blum TG et al (2020) ESR/ERS statement paper on lung cancer screening. Eur Radiol 30:3277–3294 32052170 10.1007/s00330-020-06727-7

[17] Pinsky PF, Gierada DS, Black W et al (2015) Performance of Lung-RADS in the National Lung Screening Trial: a retrospective assessment. Ann Intern Med 162:485–491 25664444 10.7326/M14-2086 PMC4705835

[18] Kramer BS, Berg CD, Aberle DR, Prorok PC (2011) Lung cancer screening with low-dose helical CT: results from the National Lung Screening Trial (NLST). J Med Screen 18:109–111 22045816 10.1258/jms.2011.011055 PMC3204895

[19] Gohagan JK, Marcus PM, Fagerstrom RM et al (2005) Final results of the Lung Screening Study, a randomized feasibility study of spiral CT versus chest X-ray screening for lung cancer. Lung Cancer 47:9–15 15603850 10.1016/j.lungcan.2004.06.007

[20] Henschke CI, McCauley DI, Yankelevitz DF et al (1999) Early Lung Cancer Action Project: overall design and findings from baseline screening. Lancet 354:99–105 10408484 10.1016/S0140-6736(99)06093-6

[21] Henschke CI, Naidich DP, Yankelevitz DF et al (2001) Early lung cancer action project: initial findings on repeat screening. Cancer 92:153–159 11443621 10.1002/1097-0142(20010701)92:1<153::aid-cncr1303>3.0.co;2-s

[22] International Early Lung Cancer Action Program (2025) I-ELCAP Protocols. Available via http://www.ielcap.org/protocols. Accessed 6 May 2025

[23] American College of Radiology (2025) Lung Imaging Reporting and Data System (Lung-RADS). Available via https://www.acr.org/Clinical-Resources/Clinical-Tools-and-Reference/Reporting-and-Data-Systems/Lung-RADS. Accessed 6 May 2025

[24] McKee BJ, Regis SM, McKee AB, Flacke S, Wald C (2015) Performance of ACR Lung-RADS in a clinical CT lung screening program. J Am Coll Radiol 12:273–276 25176499 10.1016/j.jacr.2014.08.004

[25] Henschke CI, Yip R, Ma T et al (2019) CT screening for lung cancer: comparison of three baseline screening protocols. Eur Radiol 29:5217–5226 30511179 10.1007/s00330-018-5857-5

[26] Croatian Institute of Public Health (2024) Primary Care Bulletin 2023/2. Available via https://www.hzjz.hr/wp-content/uploads/2024/05/Bilten_Obiteljska-opca-medicina_2023-2.pdf. Accessed 6 May 2025

[27] American Association of Physicists in Medicine (2025) Lung Cancer Screening CT Protocols. Available via https://aapm.org/pubs/CTProtocols/documents/LungCancerScreeningCT.pdf. Accessed 6 May 2025

